# Rat Models of Diet-Induced Obesity and High Fat/Low Dose Streptozotocin Type 2 Diabetes: Effect of Reversal of High Fat Diet Compared to Treatment with Enalapril or Menhaden Oil on Glucose Utilization and Neuropathic Endpoints

**DOI:** 10.1155/2015/307285

**Published:** 2015-07-02

**Authors:** Amey Holmes, Lawrence J. Coppey, Eric P. Davidson, Mark A. Yorek

**Affiliations:** ^1^Department of Veterans Affairs Iowa City Health Care System, Iowa City, IA 52246, USA; ^2^Department of Internal Medicine, University of Iowa, Iowa City, IA 52242, USA; ^3^Fraternal Order of Eagles Diabetes Research Center, University of Iowa, Iowa City, IA 52242, USA

## Abstract

We examined whether reversal of high fat diet, stimulating weight loss, compared to two treatments previously shown to have beneficial effects, could improve glucose utilization and peripheral neuropathy in animal models of obesity and type 2 diabetes. Rats were fed a high fat diet and treated with a low dose of streptozotocin to create models of diet induced obesity or type 2 diabetes, respectively. Afterwards, rats were transferred to a normal diet or treated with enalapril or dietary enrichment with menhaden oil for 12 weeks. Obesity and to a greater extent type 2 diabetes were associated with impaired glucose utilization and peripheral neuropathy. Placing obese rats on a normal diet improved glucose utilization. Steatosis but not peripheral neuropathy was improved after placing obese or diabetic rats on a normal diet. Treating obese and diabetic rats with enalapril or a menhaden oil enriched diet generally improved peripheral neuropathy endpoints. In summary, dietary improvement with weight loss in obese or type 2 diabetic rats was not sufficient to correct peripheral neuropathy. These results further stress the need for discovery of a comprehensive treatment for peripheral neuropathy.

## 1. Introduction

The goal of these studies was to determine whether replacing the high fat diet with a normal diet would improve glucose utilization and/or peripheral neuropathy in diet induced obese or type 2 diabetic rats. Efficacy of reversal of the high fat diet on these endpoints was compared to two distinct treatments that had previously been found to have beneficial effects: angiotensin converting enzyme inhibitor, enalapril, and dietary enrichment with omega-3 (n-3) polyunsaturated fatty acids derived from menhaden (fish) oil a natural source of eicosapentaenoic acid and docosahexaenoic acid [1–17]. The two rat models used in these studies were the diet induced obese and type 2 diabetic rat models. We previously demonstrated that diet induced obese rats develop whole body insulin resistance and sensory neuropathy associated with reduced sensory nerve conduction velocity, thermal hypoalgesia, and decreased intraepidermal nerve fiber density in the skin of the hindpaw [18]. To model type 2 diabetes we used the high fat fed/low dose streptozotocin treated rat model. We previously characterized the progression of neuropathic deficits in this model that included severe insulin resistance compared to diet induced obesity rats and a decrease in motor and sensory nerve conduction velocity as well as deficits associated with thermal nociception [19–21]. Rats fed a high fat diet do not become hyperglycemic, presumably due to compensatory hyperinsulinemia [21]. However, treating diet induced obese rats with a low dose of streptozotocin damages insulin producing *β*-cells so that hyperglycemia develops even though insulin levels are similar or even higher than in normal fed control rats [19, 21]. The diabetes in these rats is analogous to the development of human type 2 diabetes when the decline in hyperinsulinemia is not able to compensate for insulin resistance and results in the development of hyperglycemia [19]. In our hands this rat models late stage type 2 diabetes [21].

Obesity is considered to be a contributing factor to insulin resistance and type 2 diabetes [22–25]. The question being addressed is whether replacing the high fat diet of a rat model with diet induced obesity or type 2 diabetes with a normal diet would improve insulin resistance and/or peripheral neuropathy. It has been shown that humans and animal models of prediabetes and insulin resistance have sensory neuropathy like deficits [1, 21, 26–29]. Results from dietary correction were compared to the effect of treatment with enalapril or enrichment of the diet with menhaden oil. In previous studies we have found that both enalapril and menhaden oil enrichment are effective treatments for obesity and/or diabetes related neural deficits [1–5, 15, 21].

## 2. Materials and Methods

Unless stated otherwise all chemicals used in these studies were obtained from Sigma Chemical Co. (St. Louis, MO).

### 2.1. Animals

Male Sprague-Dawley (Harlan Sprague Dawley, Indianapolis, IN) rats 10-11 weeks of age were housed in a certified animal care facility and food (Harlan Teklad, #7001, Madison, WI) and water were provided ad libitum. All institutional (approval ACURF #1290202) and NIH guidelines for use of animals were followed. All possible steps were taken to avoid animal suffering during the course of these experiments. At 12 weeks of age rats were separated into nine groups. Eight of these groups were placed on a high fat diet (D12451 (45% kcal as fat, 4.7 kcal/g); Research Diets, New Brunswick, NJ), which contained 24 gm% fat, 24 gm% protein. and 41 gm% carbohydrate with the primary source of the increased fat content being lard. The remaining group was maintained on a normal diet (Harlan Teklad, #7001, 3.0 kcal/g, Madison, WI), which contained 4.25 gm% fat. Rats were maintained on the high fat diet for 8 weeks. Afterwards, four of the high fat fed group of rats were treated with streptozotocin (30 mg/kg in 0.1 M citric acid buffer, pH 4.5, i.p.). Diabetes was verified 96 h later by evaluating blood glucose levels with the use of glucose-oxidase reagent strips (Accu-Chek, Roche Inc., Indianapolis, IN). Rats having blood glucose level of 300 mg/dl (11.1 mM) or greater were considered to be diabetic. These rats as well as the four groups of high fat fed rats that were not treated with streptozotocin were maintained on the high fat diet for an additional 4 weeks. Afterwards, one of each of the four groups of high fat fed rats and diabetic rats were placed on the normal diet (obese and diabetic reversal groups), treated with enalapril (500 mg/kg in the high fat diet, obese and diabetic enalapril groups) or treated with menhaden oil by replacing 50% of the fat derived from lard in the high fat diet with menhaden oil (obese and diabetic menhaden oil groups) [1, 30]. The other group of high fat fed rats and diabetic rats remained on the standard high fat diet. At the time treatments were started there was no statistical difference between the amount of high fat diet consumed by the diet-induced obese rats and high fat fed rats treated with a low dose of streptozotocin. The high fat fed rats consumed 39 ± 5 g/day/kg rat and the diabetic rats consumed 45 ± 4 g/day/kg rat. The amount of diet consumed did not change when enalapril was added to the high fat diet or when the high fat diet was enriched with menhaden oil. Therefore, for these studies the diet-induced obese rats and diabetic rats received about the same amount of treatment.

### 2.2. Glucose Tolerance and Insulin Stimulated Glucose Uptake by Isolated Soleus Muscle

Glucose tolerance was determined by injecting rats with a saline solution containing 2 g/kg glucose, i.p., after an overnight fast [1, 21]. Rats were briefly anesthetized with isoflurane and the glucose solution was injected. Immediately prior to the glucose injection and at 15, 30, 45, 60, 120, 180, and 240 min blood samples from the tip of the tail were taken to measure circulating glucose levels using glucose-oxidase reagent strips.

### 2.3. Thermal Nociceptive Response

Thermal nociceptive response in the hindpaw was measured using the Hargreaves method as previously described [1, 21]. Briefly, the rat was placed in the observation chamber on top of the thermal testing apparatus and allowed to acclimate to the warmed glass surface (30°C) and surroundings for a period of 15 min. The mobile heat source was maneuvered so that it was under the heel of the hindpaw and then activated, a process that activates a timer and locally warms the glass surface; when the rat withdrew its paw, the timer and the heat source were turned off and the time was recorded. The heat source goes off by default after 25 sec to avoid injury to the rat. Following an initial recording, which was discarded, two measurements were made for each hindpaw, with a rest period of 5 min between each measurement. The mean of the measurements reported in sec were used as the thermal nociceptive response.

### 2.4. Motor and Sensory Nerve Conduction Velocity

On the day of terminal studies rats were weighed and anesthetized with Nembutal i.p. (50 mg/kg, i.p., Abbott Laboratories, North Chicago, IL). Motor nerve conduction velocity was determined as previously described using a noninvasive procedure in the sciatic-posterior tibial conducting system [1, 21]. The left sciatic nerve was stimulated first at the sciatic notch and then at the Achilles tendon. Stimulation consisted of single 0.2-ms supramaximal (8 V) pulses through a bipolar electrode (Grass S44 Stimulator, Grass Medical Instruments, Quincy, MA). The evoked potentials were recorded from the interosseous muscle with a unipolar platinum electrode and displayed on a digital storage oscilloscope (model 54600A, Hewlett Packard, Rolling Meadows, IL). Motor nerve conduction velocity was calculated by subtracting the distal from the proximal latency measured in milliseconds from the stimulus artifact of the take-off of the evoked potential and the difference was divided into the distance between the 2 stimulating electrodes measured in millimeters using a Vernier caliper. Sensory nerve conduction velocity was determined using the digital nerve as described [1, 21]. Briefly, hind limb sensory nerve conduction velocity was recorded in the digital nerve to the second toe by stimulating with a square-wave pulse of 0.05-ms duration using the smallest intensity current that resulted in a maximal amplitude response. The sensory nerve action potential was recorded behind the medial malleolus. Eight responses were averaged to obtain the position of the negative peak. The maximal sensory nerve conduction velocity was calculated by measuring the latency to the onset/peak of the initial negative deflection and the distance between stimulating and recording electrodes. The motor and sensory nerve conduction velocity was reported in meters per second.

### 2.5. Intraepidermal Nerve Fiber Density in the Hindpaw

Immunoreactive intraepidermal nerve fiber profiles, which are primarily sensory nerves, were visualized using confocal microscopy. Samples of skin of the right hindpaw were fixed, dehydrated, and embedded in paraffin. Sections (7 *μ*m) were collected and immunostained with anti-PGP9.5 antibody (rabbit anti human, AbD Serotic, Morpho Sys US Inc., Raleigh, NC) overnight followed by treatment with secondary antibody Alexa Fluor 546 goat anti-rabbit (Invitrogen, Eugene, OR). Profiles were counted by two individual investigators that were masked to the identity of the sample. All immunoreactive profiles within the epidermis were counted and normalized to epidermal length [1, 21, 31].

### 2.6. Physiological Markers

Nonfasting blood glucose was determined. Hemoglobin A_1_C levels were determined using a Glyco-tek affinity column kit (Helena Laboratories, Beaumont, TX). Serum samples were collected for determination of free fatty acid, triglyceride, free cholesterol, leptin, and insulin using commercial kits from Roche Diagnostics, Mannheim, Germany (for free fatty acids); Sigma Chemical Co., St. Louis, MO (for triglycerides); Bio Vision, Mountain View, CA (for cholesterol); ALPCO, Salem, NH (for leptin); EMD Millipore Corp., Billerica, MA (for insulin). The left epididymal was isolated and weight determined. To examine steatosis, liver samples were frozen in OCT compound (Sakura FineTek USA, Torrance, CA) in liquid nitrogen. Liver sections, 5 *μ*m, were incubated with BODIPY (Molecular Probes, Carlsbad, CA, USA), at a 1 : 5000 dilution in 1% BSA for 1 h at room temperature. After washing, liver sections were mounted using ProLong Gold antifade reagent (Molecular Probes, Carlsbad, CA, USA) and covered with a glass coverslip. Images were collected using Zeiss 710 LSM confocal laser scanning microscope. Images were analyzed for % area fraction of lipid droplets using ImageJ software [1, 21].

### 2.7. Data Analysis

Results are presented as mean ± S.E.M. Comparisons between the treatment groups and control and nontreated high fat fed and diabetic rats were conducted using one-way ANOVA and Bonferroni posttest comparison (Prism software; GraphPad, San Diego, CA). A *P* value of less than 0.05 was considered significant.

## 3. Results

Data in Table 1 demonstrate that the beginning weight of all the rats in the study were statistically the same. Diet induced obese rats weighed significantly more than control rats after 24 weeks on the high fat diet. Treating diet induced obese rats with enalapril or placing them on a normal diet for 12 weeks, after 12 weeks of the high fat diet, resulted in diet induced obese rats weighing about the same as control rats at the end of the study. In contrast, the weight of diet induced obese rats treated with a menhaden oil enriched diet was similar to nontreated diet induced obese rats. Diabetic rats and diabetic rats placed on the menhaden oil enriched diet weighed about the same as the control rats at the end of the study. Diabetic rats placed on the normal diet or treated with enalapril trended to weigh less than the untreated diabetic rats. We have previously reported that diet-induced obese rats and rats made diabetic by feeding a high fat diet followed by a low dose on streptozotocin trend to weigh less than when treated with enalapril compared to their untreated counterparts [1, 32]. The reason for this is unknown but could be due to an improved metabolic status in enalapril treated rats. All diabetic rats were hyperglycemic and this was not affected by treatments. Steatosis was significantly increased in diet induced obese rats and diabetic rats compared to control rats. Treating diet induced obese rats or diabetic rats by placing them on the normal diet or to a lesser extent by treating those with enalapril or menhaden oil enriched diet reduced the fatty liver condition. Weight of the left epididymal was significantly increased in diet induced obese rats. Placing diet induced obese rats on a normal diet or treating them with enalapril but not menhaden oil corrected the left epididymal fat pad weight toward control levels. The weight of the left epididymal fat in diabetic rats was similar to control.

**Figure 1:**
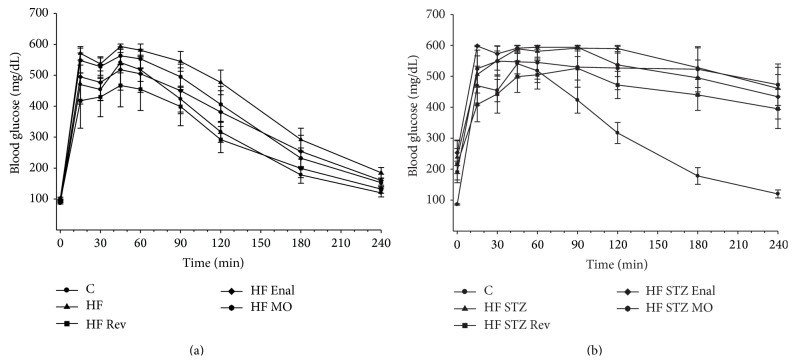
Effect of treatment of diet induced obese rats (a) and high fat fed/low dose streptozotocin type 2 diabetic rats (b) with normal diet, enalapril, or menhaden oil on glucose tolerance. Glucose tolerance was determined as described in Section 2. Data are presented as the mean ± S.E.M. in mg/dl. The number of rats in each group was the same as shown in Table 1. Control rats (C), diet induced obese rats (HF), diabetic rats (HF STZ), and rats returned to a normal diet or treated with enalapril or placed on a menhaden oil enriched diet designated as Rev, Enal, or MO, respectively.

Figure 1 demonstrates that diet induced obese rats (Figure 1(a), area under the curve (AUC): 133% of control,* P* < 0.05) and to a greater extent diabetic rats (Figure 1(b), AUC: 171% of control,* P* < 0.05) had significantly impaired glucose clearance compared to control rats. After 12 weeks on a high fat diet, high fat fed rats placed on a normal diet for 12 weeks had completely normalized glucose clearance (Figure 1(a), AUC: 95% of control,* P* < 0.05 compared to diet induced obese rats). Treating high fat fed rats with enalapril or menhaden oil enriched diet also improved glucose clearance compared to diet induced obese rats but was not as efficacious as replacing the high fat diet with a normal diet (Figure 1(a), AUC: 113% and 118% of control, resp.). Placing diabetic rats on a normal diet for 12 weeks, after 12 weeks of the high fat diet and 4 weeks of hyperglycemia, had a modest effect on glucose clearance (Figure 1(b), AUC: 144% of control,* P* < 0.05 compared to control rats) but the difference between diabetic rats and diabetic rats placed on the normal diet did not reach statistical significance. Treating diabetic rats with enalapril or menhaden oil enriched diet had no effect on improving glucose clearance (Figure 1(b), AUC: 167% and 163%, resp.,* P* < 0.05 compared to control rats).

Data in Table 2 demonstrate that feeding rats a high fat diet to induce obesity or subsequent treatment by placing obese rats on a normal diet or treating them with enalapril or menhaden oil enriched diet did not significantly change serum levels of free fatty acids, triglycerides, or cholesterol. In diabetic rats, serum free fatty acid, triglyceride, and cholesterol levels were all significantly increased compared to control rats. Placing diabetic rats on a normal diet or treating them with enalapril or menhaden oil enriched diet lowered serum lipid levels compared to untreated diabetic rats. Serum leptin levels were significantly increased in diet induced obese rats compared to control rats (Table 2). Placing diet induced obese rats on a normal diet or treating them with enalapril lowered serum leptin levels. In contrast, leptin levels remained significantly elevated when diet induced obese rats were treated with a diet enriched with menhaden oil. Leptin levels were unchanged in diabetic rats and diabetic rats placed on a normal diet or treated with enalapril or diet enriched with menhaden oil. Overall, serum leptin levels mirrored the change in the epididymal fat pad weight. This was expected since leptin is mainly produced by adipose tissue and its circulating levels correlate with the amount of body fat [33]. Serum insulin levels appeared lower in diabetic rats compared to control or diet induced obese rats but the difference was not significant. Treating diet induced obese rats with a diet containing enalapril or enriched with menhaden oil caused a significant increase in serum insulin levels compared to control or diet induced obese rats. There was a trend toward increased insulin levels in serum of diabetic rats placed on a normal diet or treated with enalapril or a diet enriched with menhaden oil. However, the difference was not significant. Therefore, one possible explanation for the beneficial effects observed with dietary improvement and/or treatment with enalapril or menhaden oil of obese and diabetic rats is increase in serum insulin levels.

Data in Table 3 demonstrate that sensory nerve conduction velocity but not motor nerve conduction velocity is significantly decreased in diet induced obese rats compared to control rats. Placing diet induced obese rats on a normal diet did not improve sensory nerve conduction velocity. In contrast, treating diet induced obese rats with enalapril and to a greater extent with a diet enriched with menhaden oil improved sensory nerve conduction velocity. Both motor and sensory nerve conduction velocity was significantly decreased in diabetic rats (Table 3). Placing diabetic rats on a normal diet did not improve motor or sensory nerve conduction velocity. Motor and sensory nerve conduction velocity was partially improved by treating diabetic rats with enalapril and to a greater extent by treating diabetic rats with a diet enriched with menhaden oil.

Sensitivity to a thermal stimulus was significantly impaired in diet induced obese rats and diabetic rats (Table 3). Placing diet induced obese rats on a normal diet and to a greater extent treating them with enalapril or with a diet enriched in menhaden oil improved thermal nociception. Placing diabetic rats on a normal diet did not improve thermal sensitivity compared to untreated diabetic rats. In contrast, thermal nociception was significantly improved when diabetic rats were treated with enalapril or with a diet enriched in menhaden oil. Intraepidermal nerve fiber density was significantly decreased in diet induced obese rats and diabetic rats compared to control rats (Table 3). Placing diet induced obese rats on a normal diet did not significantly improve intraepidermal nerve fiber density. In contrast, treating diet induced obese rats with enalapril or a diet enriched with menhaden oil did significantly improve intraepidermal nerve fiber density. Placing diabetic rats on a normal diet and to a greater extent treating diabetic rats with enalapril or a diet enriched with menhaden oil improved intraepidermal nerve fiber density.

## 4. Discussion

The major findings from this study were that replacing the high fat diet in diet induced obese rats with normal diet improved glucose utilization and reduced fatty liver. However, the neuropathic endpoints were not improved. Placing type 2 diabetic rats on a normal diet also reduced fatty liver and trended to improve glucose utilization but did not correct peripheral neuropathy. In contrast, treating diet induced obese rats or type 2 diabetic rats with enalapril and to a greater extent with a menhaden oil enriched diet slowed progression and/or improved peripheral neuropathy so that at the end of the study there was a significant difference between untreated and treated rats in most neuropathy endpoints, although these treatments had a lesser effect on improving glucose utilization.

Placing diet induced obese rats on a normal diet for 12 weeks, after 12 weeks of a high fat diet, induced weight loss and at the end of the study period these rats weighed the same as the control rats. Feeding rats a high fat diet causes a progressive impairment in vascular and neural function and after 24–32 weeks of a high fat diet there is a significant decrease in sensory nerve conduction velocity and intraepidermal nerve fiber density and thermal hypoalgesia [18]. When the diet of rats fed high fat diet for 12 weeks was replaced with a normal diet and analyses performed 12 weeks later, sensory nerve conduction velocity and intraepidermal nerve fiber density were significantly decreased. Thermal latency was increased but was not significantly different from either control or rats fed a high fat diet for 24 weeks. This suggests that even after replacing the high fat diet with a normal diet prior to a significant decrease in sensory nerve endpoints, there is no notable improvement in peripheral neuropathy even though glucose utilization and steatosis were corrected [18]. Since for some of the neural endpoints examined there was a trend toward improvement when diet-induced obese and diabetic rats were placed on a normal diet it is possible that neural function would have improved further if the normal diet was allowed to continue for a longer duration. Nonetheless, these results demonstrate that any recovery in neural function is a slow process.

In obese patients with type 2 diabetes a year after having undergone gastric bypass surgery there was a significant improvement in weight, blood glucose, hemoglobin A_1_C, and insulin resistance and the percentage of patients with neuropathy was lower than the number of cases at baseline [34]. Management of weight and physical activity has been shown to prevent or delay the development of type 2 diabetes [35, 36]. This study did not examine the combined effects of weight loss with exercise but it has been shown that exercise can increase the cutaneous nerve density in diabetic patients without neuropathy and reinnervation capacity in patients with metabolic syndrome [37, 38]. Others have shown that moderate aerobic exercise can help disrupt the progression of peripheral neuropathy in type 2 diabetic patients but animal studies suggest that exercise alone cannot prevent peripheral nerve damage from hyperglycemia [39, 40].

Our study demonstrated that the progression of the effect of a high fat diet on neurological endpoints can be slowed or perhaps improved by treating diet induced obese rats after 12 weeks on a high fat diet. Treating diet-induced obese rats with a high fat diet containing enalapril or enriched with menhaden oil normalized sensory nerve conduction velocity and intraepidermal nerve fiber density and sensitivity [32].

Once hyperglycemia occurs reversal of neurological deficits is more difficult [41]. In this study placing type 2 diabetic rats on a normal diet for 12 weeks after 12 weeks of a high fat diet with 4 weeks of hyperglycemia corrected steatosis, improved glucose utilization and serum lipid levels. However, there was little improvement in neurological endpoints. As previously reported, treating type 2 diabetic rats with a high fat diet containing enalapril and to a greater extent with a high fat diet enriched with menhaden oil slowed the progression or improved diabetic neuropathy endpoints [1, 30]. In previous studies we have shown that treating diet-induced obese rats with enalapril or diabetic rodents with enalapril or menhaden oil reduces oxidative and inflammatory stress and menhaden oil is a source of production of n-3 fatty acid metabolites that have neuroprotective properties [1, 4, 5, 15–17, 30, 32, 42]. Replacing the high fat diet with a normal diet was more effective than treating type 2 diabetic rats with enalapril or menhaden oil on improving glucose utilization and normal diet replacement or treatment with enalapril was more effective than menhaden oil in improving steatosis. Thus, from this study it would seem that weight loss alone through dietary management is insufficient in delaying the progression of diabetic neuropathy.

## 5. Conclusions

Complications linked to diet induced obesity such as impaired glucose utilization and fatty liver can be improved by reducing fat consumption and inducing weight loss. However, this approach is less effective in improving neural deficits. Once hyperglycemia has developed reducing fat intake through the diet was found to still improve steatosis but was less effective in improving glucose utilization and diabetic peripheral neuropathy. This suggests that an effective treatment for peripheral neuropathy associated with either prediabetes or diabetes is needed and to be efficacious will likely require early detection followed by a combination of lifestyle changes, diet management, and treatments designed to counteract the effects of hyperglycemia and hyperlipidemia.

## Figures and Tables

**Table 1:** Effect of reversal of high fat diet, enalapril, or menhaden oil in diet-induced obese or type 2 diabetic rats on change in body weight, nonfasting blood glucose, hemoglobin A_1_C, steatosis, and epididymal fat pad.

Condition	Start weight (g)	End weight (g)	Blood glucose (mg/dL)	Hb A_1_C (%)	Steatosis (% area)	Epididymal fat pad (g)
Control (12)	326 ± 3	491 ± 9	149 ± 10	6.6 ± 0.4	3.0 ± 0.5	2.8 ± 0.1
Obese (11)	329 ± 3	560 ± 18^a^	144 ± 6	7.0 ± 0.7	5.4 ± 0.7^a^	5.9 ± 0.6^a^
Obese + Normal diet (10)	320 ± 3	496 ± 11^d^	133 ± 5	7.8 ± 0.8	2.7 ± 0.3^d^	3.1 ± 0.2^d^
Obese + enalapril (12)	311 ± 1	489 ± 12^d^	149 ± 4	6.3 ± 0.4	4.1 ± 0.6	3.3 ± 0.3^d^
Obese + menhaden oil (12)	312 ± 2	585 ± 12^a^	152 ± 9	5.8 ± 0.5	4.0 ± 0.5	5.8 ± 0.4^a^

Diabetic (10)	325 ± 4	493 ± 24	382 ± 20^a,d^	14.6 ± 1.0^a,d^	7.7 ± 1.2^a^	2.8 ± 0.3^d^
Diabetic + normal diet (10)	321 ± 3	413 ± 21^b^	418 ± 25^a,b,d^	11.9 ± 2.0^a,b,d^	3.1 ± 0.5^c^	1.6 ± 0.3^b^
Diabetic + enalapril (11)	315 ± 3	416 ± 9^b,c^	415 ± 23^a,b,d^	11.9 ± 1.6^a,b,d^	3.4 ± 0.8^c^	2.5 ± 0.1^d^
Diabetic + menhaden oil (11)	310 ± 4	450 ± 23^b^	364 ± 21^a,b,d^	9.5 ± 1.0^a,b,c,d^	5.2 ± 0.8	3.3 ± 0.5^b,d^

Data are presented as the mean ± S.E.M. ^a^ *P* < 0.05 compared to control; ^b^ *P* < 0.05 compared to obese matched condition; ^c^ *P* < 0.05 compared to diabetic; ^d^ *P* < 0.05 compared to obese. Parentheses indicate the number of experimental animals.

**Table 2:** Effect of reversal of high fat diet, enalapril, or menhaden oil in diet-induced obese or type 2 diabetic rats on nonfasting serum free fatty acids, triglycerides, cholesterol, leptin, and insulin levels.

Condition	Free fatty acids (mmol/L)	Triglycerides (mg/dL)	Cholesterol (mg/mL)	Leptin (ng/mL)	Insulin (ng/mL)
Control (12)	0.20 ± 0.03	85 ± 8	3.0 ± 0.5	11.1 ± 1.1	4.3 ± 0.8
Obese (11)	0.20 ± 0.05	94 ± 8	4.1 ± 0.3	38.9 ± 5.8^a^	4.5 ± 0.8
Obese + normal diet (10)	0.16 ± 0.03	94 ± 13	3.6 ± 0.5	10.4 ± 0.8^d^	3.8 ± 0.7
Obese + enalapril (12)	0.20 ± 0.02	83 ± 12	3.4 ± 0.3	24.8 ± 4.1	29.0 ± 2.6^a,c,d^
Obese + menhaden oil (12)	0.24 ± 0.02	62 ± 9	3.7 ± 0.6	65.6 ± 17.4^a^	19.5 ± 3.2^a,c,d^

Diabetic (10)	0.63 ± 0.11^a,d^	319 ± 59^a,d^	8.0 ± 2.3^a^	16.7 ± 8.5	0.9 ± 0.4
Diabetic + normal diet (10)	0.31 ± 0.06	232 ± 46	6.0 ± 1.0	5.2 ± 1.9	5.2 ± 1.4
Diabetic + enalapril (11)	0.48 ± 0.15	179 ± 36	3.2 ± 0.5	4.3 ± 1.2	6.7 ± 1.5^b^
Diabetic + menhaden oil (11)	0.29 ± 0.03	104 ± 22^c^	4.4 ± 0.7	16.3 ± 4.0^b^	5.5 ± 1.3^b^

Data are presented as the mean ± S.E.M. ^a^ *P* < 0.05 compared to control; ^b^ *P* < 0.05 compared to obese matched condition; ^c^ *P* < 0.05 compared to diabetic; ^d^ *P* < 0.05 compared to obese. Parentheses indicate the number of experimental animals.

**Table 3:** Effect of reversal of high fat diet, enalapril, or menhaden oil in diet-induced obese or type 2 diabetic rats on motor and sensory nerve conduction velocity, thermal nociception, and intraepidermal nerve fiber density.

Condition	MNCV (m/sec)	SNCV (m/sec)	Thermal nociception (sec)	Intraepidermal nerve fiber (profiles/mm)
Control (12)	61.3 ± 1.3	21.0 ± 0.4	10.0 ± 0.6	15.1 ± 0.5
Obese (11)	60.4 ± 2.3	18.5 ± 0.4^a^	15.5 ± 0.7^a^	10.3 ± 0.7^a^
Obese + normal diet (10)	58.1 ± 2.2	19.1 ± 0.5^a^	13.4 ± 1.0	11.6 ± 1.0^a^
Obese + enalapril (12)	60.2 ± 2.6	19.7 ± 0.3	11.9 ± 0.6^d^	14.1 ± 0.7^d^
Obese + menhaden oil (12)	64.3 ± 2.9	20.4 ± 0.3^d^	11.3 ± 0.7^d^	14.1 ± 0.4^d^

Diabetic (10)	44.3 ± 1.6^a,d^	17.3 ± 0.3^a^	17.4 ± 1.0^a^	10.7 ± 0.9^a^
Diabetic + normal diet (10)	47.3 ± 1.4^a,b,d^	18.6 ± 0.4^a^	15.9 ± 0.9^a^	12.8 ± 0.8
Diabetic + enalapril (11)	48.5 ± 1.8^a,b^	19.0 ± 0.5	11.9 ± 0.8^c^	14.1 ± 0.9^c^
Diabetic + menhaden oil (11)	52.3 ± 1.6^b,c^	20.0 ± 0.4^c^	10.6 ± 0.6^c^	14.6 ± 0.9^c^

Data are presented as the mean ± S.E.M. ^a^ *P* < 0.05 compared to control; ^b^ *P* < 0.05 compared to obese matched condition; ^c^ *P* < 0.05 compared to diabetic; ^d^ *P* < 0.05 compared to obese. Parentheses indicate the number of experimental animals.
